# Management of heavy menstrual bleeding in a multidisciplinary young women’s clinic: a Dutch experience

**DOI:** 10.1016/j.rpth.2025.102959

**Published:** 2025-06-27

**Authors:** Yara Dixon, Bart Kabboord, Carla Groenestein-Sondaal, Marieke J.H.A. Kruip, Carolien van der Velden, Greta Mulders, C. Heleen van Ommen

**Affiliations:** 1 Department of Pediatric Hematology, Erasmus University Medical Centre, Rotterdam, the Netherlands; 2 Department of Gynecology, Erasmus Medical Center, Rotterdam, the Netherlands; 3 Department of Hematology, Erasmus University Medical Center, Rotterdam, the Netherlands; 4 Department of Pediatric Hematology & Oncology, Erasmus Medical Center Sophia Children’s Hospital, Rotterdam, the Netherlands

**Keywords:** hemorrhagic disorders, iron deficiency, menorrhagia, oral contraceptives, tranexamic acid, von Willebrand disease

## Abstract

**Background:**

Heavy menstrual bleeding (HMB) is common in young women. It may lead to iron deficiency with or without anemia, decreased school, work, sport, and social participation, and may be the first sign of underlying bleeding disorders (BDs).

**Objectives:**

To study the prevalence of BDs, initial and effective management of HMB and iron deficiency (anemia) in young women referred to a tertiary multidisciplinary HMB clinic.

**Methods:**

This was a retrospective, single-center chart review evaluating patients aged ≤25 years who visited the multidisciplinary HMB clinic between March 2018 and December 2023.

**Results:**

In total, 200 patients (median age, 15 years) were included. At first consultation, BD was already diagnosed in 47 (24%) patients, particularly von Willebrand disease (*n* = 27). In 32 of 153 (21%) remaining patients, a new BD was diagnosed (von Willebrand disease, *n* = 21; platelet aggregation defect, *n* = 8). Initial therapy of HMB mainly consisted of tranexamic acid with (*n* = 52, 26%) or without (*n* = 50, 30%) combined oral contraceptive pill. In 60 patients (30%), initial therapy was effective. Effective HMB management was mostly achieved with tranexamic acid combined with a combined oral contraceptive pill (*n* = 75, 38%) after a median of 1 treatment change (range, 0-10). Iron deficiency was present in 52% of patients, with approximately half having anemia. Treatment consisted of oral and/or intravenous iron supplementation in 77 patients and red blood cell transfusion in 9 patients.

**Conclusion:**

BDs and iron deficiency (anemia) were common in young women with HMB. Multiple treatment strategies were needed to achieve an acceptable outcome. A multidisciplinary approach may offer complementary expertise in this patient group.

## Essentials

• HMB is common and can impact daily life and overall well-being.
• This study reviewed young women with HMB at a specialized clinic in the Netherlands.
• About 20% had an undiagnosed bleeding disorder, with von Willebrand disease most common.
• A mix of tranexamic acid and hormonal therapy was most effective for managing HMB symptoms.

## Graphical abstract

**Figure:**
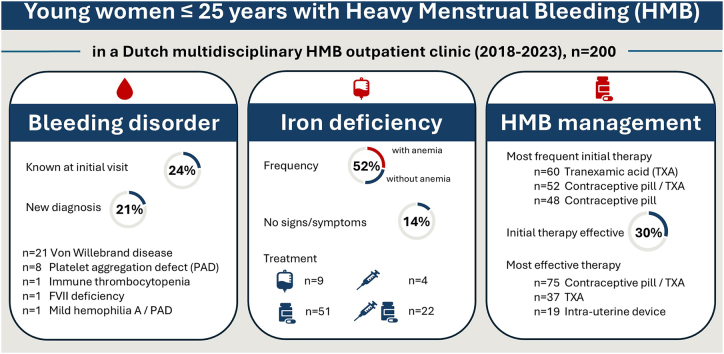

## 1 Introduction

Heavy menstrual bleeding (HMB) has traditionally been defined as blood loss > 80 mL per cycle or menstrual periods lasting >7 days [1]. However, the entity of HMB has been best defined by the United Kingdom National Institute for Health and Care Excellence as excessive menstrual blood loss, which interferes with the woman’s physical, social, emotional, and/or material quality of life [2]. HMB affects more than a quarter of women in the general population and about one-third of female adolescents [3,4].

The etiology of HMB can be categorized using the PALM-COEIN system [5]. Structural causes include Polyps, Adenomyosis, Leiomyoma, and Malignancy, while nonstructural causes encompass Coagulopathy, Ovulatory dysfunction, Endometrial disorders, Iatrogenic factors, and uNclassified conditions. In adolescents, the most common cause is immaturity of the hypothalamic-pituitary–ovarian axis, leading to anovulatory cycles [6]. Additionally, HMB may indicate an underlying inherited bleeding disorder, such as von Willebrand disease (VWD) or platelet aggregation defects [[6], [7], [8]]. A recent systematic review of 41,541 patients reported an overall bleeding disorder prevalence of 30%, with age-dependent variations: 16% in adults with HMB and 39% in adolescents (12-18 years) [6].

Besides the identification of a bleeding disorder, the concomitant presence of iron deficiency, which may be present without anemia, is an important clinical finding in many women with HMB [9]. Iron is crucial for numerous functions, including DNA synthesis and repair, enzymatic activity, mitochondrial function, and neurotransmitter production and function [10]. Iron deficiency is associated with fatigue, restlessness, behavioral issues, and cognitive disorders [11,12]. Consequently, iron supplementation has been shown to significantly improve muscle fatigability in iron-depleted, nonanemic women, as well as cognition, particularly verbal learning and memory, in nonanemic, iron-deficient adolescent girls [11,12]. A meta-analysis of 14 randomized trials further demonstrated that iron supplementation enhances attention and concentration in nonanemic iron-deficient children aged >6 years, adolescents, and premenopausal women [13].

For optimal management of HMB, a multidisciplinary approach is required [[14], [15], [16]]. The aims of HMB treatment are to reduce menstrual blood loss to a level that does not interfere with woman’s physical, social, emotional, and/or material quality of life, prevent and treat iron deficiency, check for unknown bleeding disorders (BDs), treat menstrual pain, provide education, and improve quality of life. Therefore, the Hemophilia Treatment Center of the Erasmus University Medical Center started an outpatient clinic for young women aged ≤25 years with HMB in March 2018. The team consists of a pediatric hematologist, a hematologist, a gynecologist, and a pediatric and adult nurse practitioner. In the current study, we evaluated the prevalence of BDs, as well as the initial and effective management of HMB in patients who visited the HMB clinic since its kickoff in March 2018. Furthermore, we studied the prevalence and treatment of iron deficiency (anemia).

## 2 Methods

This was a retrospective, single-center chart review conducted in the outpatient HMB clinic of the Erasmus University Medical Center in Rotterdam, the Netherlands. Postmenarche adolescents and young women aged ≤25 years referred to the HMB clinic between March 1, 2018, and December 31, 2023, were included in the study. Patients were excluded if they (1) were aged >25 years, (2) did not have diagnosis of HMB, or (3) wished to become pregnant. Follow-up of all patients continued until effective management was achieved, as of December 31, 2024, or due to loss to follow-up. Due to the retrospective design of this study, the Medical Research Involving Human Subjects Act did not apply to this study, as stated by our Medical Ethics Committee (MEC-2020-0903, date: December 4, 2020).

### 2.1 Data collection

Data from all included patients were collected in digital case report forms and in a Statistical Package for the Social Sciences database. The following data were collected: age at first consultation, age at menarche, presence of bleeding disorder at first consultation, and period characteristics such as presence of heavy menstrual blood loss since menarche, duration of periods, period regularity, changing patterns of pads or tampons, Pictorial Blood loss Assessment Chart (PBAC) score [17] at first consultation and at follow-up, presence of flooding, clots, or pain during menstruation; family history of HMB, BDs, and gynecological reasons for HMB; International Society on Thrombosis and Haemostasis Bleeding Assessment Tool score (ISTH-BAT score) [18] and newly diagnosed bleeding disorder; initial management of HMB and effective HMB management; signs and symptoms of iron deficiency with or without anemia, including fatigue, dizziness, and headache; presence of iron deficiency and anemia and anemia treatment.

### 2.2 Laboratory testing

Laboratory testing, standardized according to a protocol developed prior to the establishment of the outpatient HMB clinic, included complete blood count, ferritin, transferrin saturation, activated partial thromboplastin time, prothrombin time, platelet function analyzer, fibrinogen, von Willebrand factor (VWF) analysis, factor (F)VII, FVIII, and FXI. Platelet aggregation testing with light transmission aggregometry (LTA; repeated if abnormal) was done if VWF analysis was normal and patient had additional bleeding symptoms besides HMB, at the discretion of the treating physician.

All coagulation studies were done at the hemostasis laboratory of the Erasmus University Medical Center. Blood sampling was performed using a Vacutainer system (Becton Dickinson) and vials containing either sodium citrate (final concentration 0.109 mol/L) or EDTA (1.8 mg/mL, Plymouth). Activated partial thromboplastin time (Dade Actin FS [Siemens]), prothrombin time (Thromborel S [Siemens]), and fibrinogen (Thrombin Reagent [Siemens]) were measured on a Sysmex CS-5100 (Siemens Healthcare Diagnostics B.V.). Collagen-adenosine diphosphate and collagen-epinephrine cartridges were used to measure closure times (seconds) on a PFA-200 (Siemens Healthineers). VWF antigen levels were determined using an in-house ELISA assaywith polyclonal rabbit antihuman VWF antibodies (Dako) for capturing and detection. VWF collagen binding was measured by an in-house ELISA assay using collagen type I for capturing (Sigma-Aldrich) and polyclonal rabbit antihuman VWF antibodies (Dako) for detection. VWF activity was measured with the VWF:GP1bM Innovance assay from Siemens Healthineers on a Sysmex CS-5100. FVIII activity (FVIII:C) was measured using a one-stage clotting assay (TriniCLOT; bioMérieux) on the Sysmex CS-5100 (Siemens Healthineers). LTA was performed on a Chrono-Log aggregometer 490 (Stago).

### 2.3 Definitions

HMB was defined by menstrual duration ≥7 days and/or bleeding through a pad or tampon or both within 2 hours. VWD was diagnosed according to the American Society of Hematology, the ISTH, the National Hemophilia Foundation, and the World Federation of Hemophilia guidelines [19]. Hemophilia carriership was based on family history (daughters of fathers with DNA-proven hemophilia) or DNA testing. In addition, hemophilia carriership and rare BDs, including FV, FVII, and FXI, were defined as described in the communications from the Scientific and Standardization Committees of the ISTH [20,21]. Platelet aggregation defects were diagnosed based on 2 consecutive abnormal LTA results demonstrating a reduced response to at least 1 consistent agonist [22]. Effective HMB management was defined as management that provided relief of HMB-related symptoms, which included changing pads or tampons >2 hours, duration of bleeding period <7 days, improvement of PBAC scores if measured, no iron deficiency, and minimal side effects of hormonal contraception if used. The effect of HMB management was evaluated after a follow-up of at least 1 month. Iron deficiency was defined as serum ferritin levels ≤ 30 ng/mL or transferrin saturation <20% [10]. Anemia was defined as hemoglobin levels < 11 g/dL in patients aged ≤19 years and < 12 g/dL in patients aged 19 to 26 years.

### 2.4 Outcomes

Outcomes of this study included the prevalence and type of BDs identified in our cohort of young women with HMB, the initial management and effectiveness of HMB treatment, assessed after at least 1 complete menstrual cycle, and the prevalence and treatment of iron deficiency with and without anemia.

### 2.5 Statistical analysis

Data were analyzed using descriptive statistics. Continuous variables were reported as median with range and compared using the Mann–Whitney *U*-test for independent groups. Categorical variables were reported as *n* (%) and compared using the chi-squared or Fisher’s exact test as applicable. Statistical significance was assumed at the 5% level. Statistical analyses were performed using Statistical Package for the Social Sciences Statistics version 25 (IBM Corporation) for Windows.

## 3 Results

### 3.1 Patients

**Figure 1:**
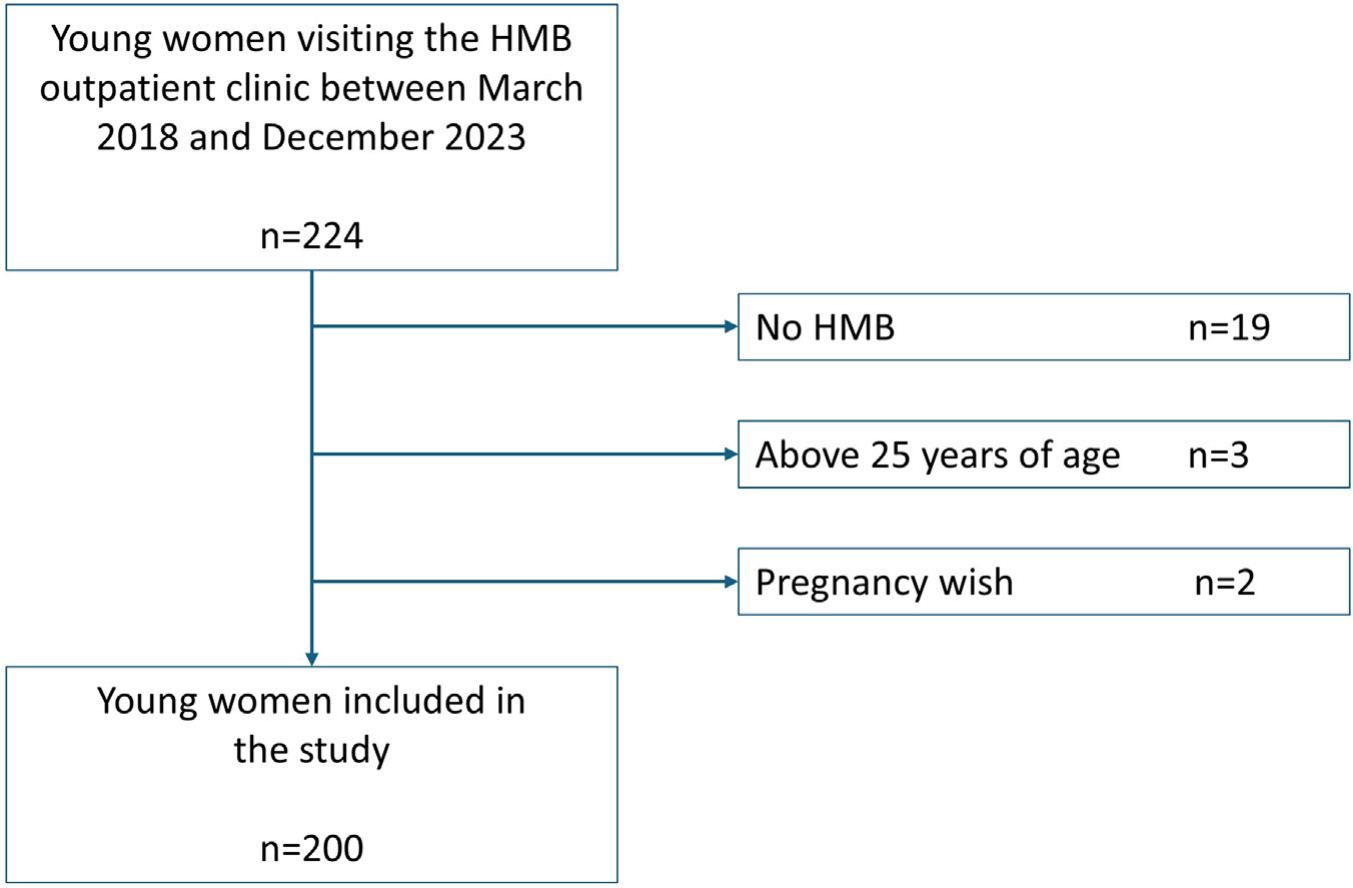
Flow diagram of young women with heavy menstrual bleeding (HMB).

A total of 224 adolescent and young women visited the outpatient HMB clinic between March 2018 and December 2023 and were considered eligible for this study. They were referred to the clinic by general physicians, secondary care pediatricians, and gynecologists. Twenty-four patients were excluded due to not meeting the HMB criteria (*n* = 19), being aged >25 years (*n* = 3), or expressing a wish for pregnancy (*n* = 2; Figure 1). The characteristics of the 200 eligible patients are summarized in Table 1. The majority of patients were aged <19 years (*n* = 189, 90%). The most commonly reported symptoms were flooding on ≥1 days (91%) and changing sanitary products at least once every 2 hours (81%). Additionally, 81 of 183 (44%), 46 of 190 (24%), and 18 of 179 (10%) patients reported a positive family history for HMB, BDs, or gynecological disorders, respectively. The median follow-up of the patients was 14 months (range, 0-113 months). Four patients were diagnosed with endometriosis.

**Table 1:** Clinical characteristics of 200 patients with heavy menstrual bleeding.

Patient characteristics	
Age (y) at first consultation, median (range)	15 (8-25)
Age (y) at menarche, median (range)	12 (8-18)
Ethnicity, *n* (%)	
White	162 (81)
African/Caribbean	10 (5)
Asian	14 (7)
Mixed	14 (7)
Known bleeding disorder at first consultation, *n* (%)	47 (24)
ISTH-BAT score (age <18 y, *n* = 180), median (range)	3 (3-11)
ISTH-BAT score (age ≥ 18 y, *n* = 20), median (range)	3 (3-8)

Period characteristics	
HMB since menarche, n/N (%)	128/179 (72)
Period length ≥7 d, n/N (%)	86/179 (48)
Irregular periods, n/N (%)	83/171 (49)
Menstruation products, *n/N* (%)	
Pads	76/153 (50)
Tampons	6/153 (4)
Pads and tampons	30/153 (20)
Maxi pads	38/153 (25)
Changing patterns ≤2 h, *n/N* (%)	120/148 (81)
Flooding ≥1 d, *n/N* (%)	152/167 (91)
Clot loss present, *n/N* (%)	91/178 (51)
Pain, *n/N* (%)	158/189 (84)
PBAC at first consultation, median (range)^a^	264 (100-1103)
PBAC at follow-up consultation 3 mo, median (range)^b^	143 (31-267)

HMB, heavy menstrual bleeding; ISTH-BAT, International Society on Thrombosis and Haemostasis Bleeding Assessment Tool; PBAC, Pictorial Blood Assessment Chart.

a In 37 patients.

b In 9 patients.

### 3.2 Bleeding Disorders

**Figure 2:**
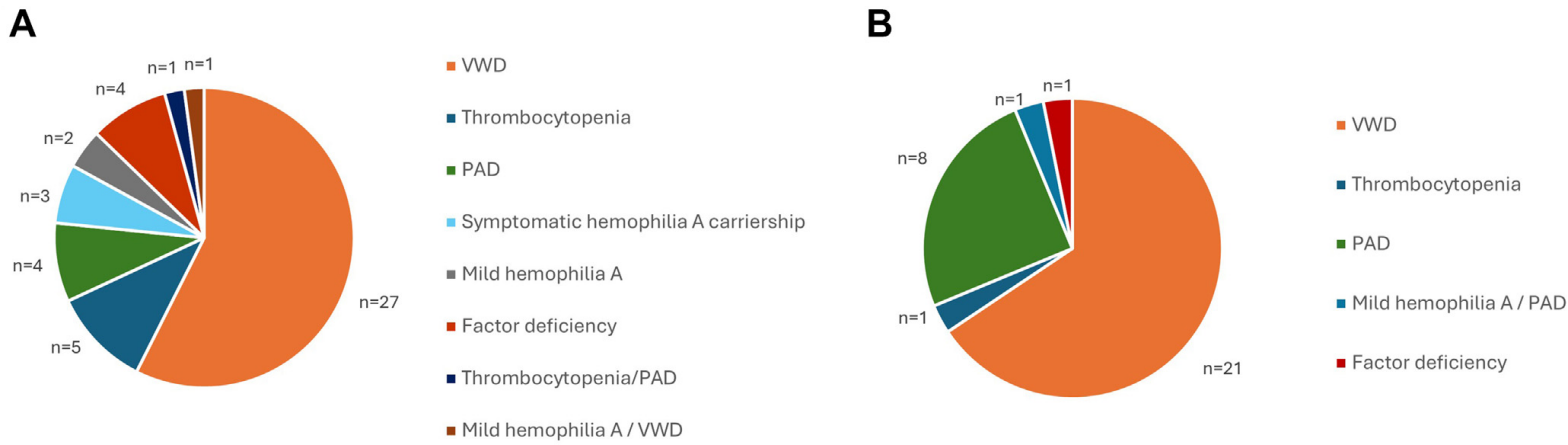
Frequency of bleeding disorders (BDs) in patients with heavy menstrual bleeding. (A) BDs were known in 47 of 200 patients at first consultation, and (B) BDs were diagnosed in 32 of remaining 153 patients. PAD, platelet aggregation defect; VWD, von Willebrand disease.

Forty-seven patients (24%) were already diagnosed with a bleeding disorder at their first consultation, including VWD (*n* = 27), immune-mediated thrombocytopenia (*n* = 5), platelet aggregation defect (*n* = 4), symptomatic carriership of hemophilia A (*n* = 3), and mild hemophilia A (*n* = 2; Figure 2A). Fifteen patients had type 1 VWD, including 4 patients with VWF levels < 0.30 IU/mL. Twelve patients had type 2 VWD. The median VWF plasma levels of patients with VWD are shown in Table 2. A platelet aggregation defect was caused by an *FLNA* gene variant in 2 siblings. FVIII:C levels of the patients with mild hemophilia A were 34 and 37 IU/mL. The FVIII:C levels of the symptomatic carriers of hemophilia A varied between 0.62 and 0.73 IU/mL. In addition to HMB, they also experienced other bleeding symptoms. Other BDs included factor deficiencies, such as deficiencies of FV (*n* = 1; FV < 0.02 IU/mL), FVII (*n* = 2; FVII 24 and 49 IU/mL, respectively), and FXI (*n* = 1; FXI 0.47 IU/mL). Two patients had a combination of BDs: mild VWD type 1 and mild hemophilia A (FVIII:C 0.15 IU/mL), and thrombocytopenia and platelet aggregation defect (*ACTN1* gene mutation). Supplementary coagulation studies in patients with an already known bleeding disorder at their first consultation revealed an additional platelet aggregation defect in 1 patient with FXI deficiency, 1 patient with mild VWD type 1, and 1 patient with symptomatic carriership of hemophilia A.

**Table 2:** Laboratory results of patients with von Willebrand disease (median, range).

	VWF:ag (IU/mL)	VWF:act (IU/mL)	VWF:CB (IU/mL)	FVIII:C (IU/mL)
**VWD already known at first consultation**				
*n* = 28 patients	0.40 (0.06-1.52)	0.34 (0.04-0.49)	0.39 (0.02-0.97)	0.62 (0.01-1.42)

**VWD newly diagnosed**				
*n* = 21 patients	0.49 (0.36-0.67)	0.48 (0.37-0.59)	0.60 (0.40-0.78)	0.86 (0.65-1.57)

FVIII:C, factor VIII activity; IU, International Units; VWD, von Willebrand disease; VWF:act, von Willebrand factor activity; VWF:ag, von Willebrand factor antigen; VWF:CB, von Willebrand factor collagen binding.

In 32 of 153 (21%) patients with HMB, who were not known to have a bleeding disorder at their first consultation, coagulation testing revealed a new bleeding disorder, including mild VWD type 1 (*n* = 21), platelet aggregation defect (*n* = 8), immune-mediated thrombocytopenia (*n* = 1), factor VII deficiency (*n* = 1; FVII 35 IU/mL) and a combination of mild hemophilia A (FVIII:C 0.28 IU/mL) and platelet aggregation defect (*n* = 1; Figure 2B). Median VWF plasma levels of patients with VWD are shown in Table 2. All patients <18 years had an ISTH-BAT score of ≥2. The median ISTH-BAT score was 3 (IQR, 1) in patients <18 years without diagnosis of new bleeding disorder, and 4 (IQR, 2) in patients <18 years with diagnosis of new bleeding disorder. An ISTH-BAT score of ≥5 was present in 13 of 32 (41%) patients <18 years with a new bleeding disorder, and in 32 of 139 (24%) patients <18 years without a new bleeding disorder (*P* = .065). In 7 of 32 (22%) patients with a new bleeding disorder, family history for BDs was positive compared with 24 of 113 (21%) patients without bleeding disorder (*P* = .9). HMB was the first symptom of a newly diagnosed bleeding disorder in 11 of 32 (34%) patients.

### 3.3 Management of HMB

**Figure 3:**
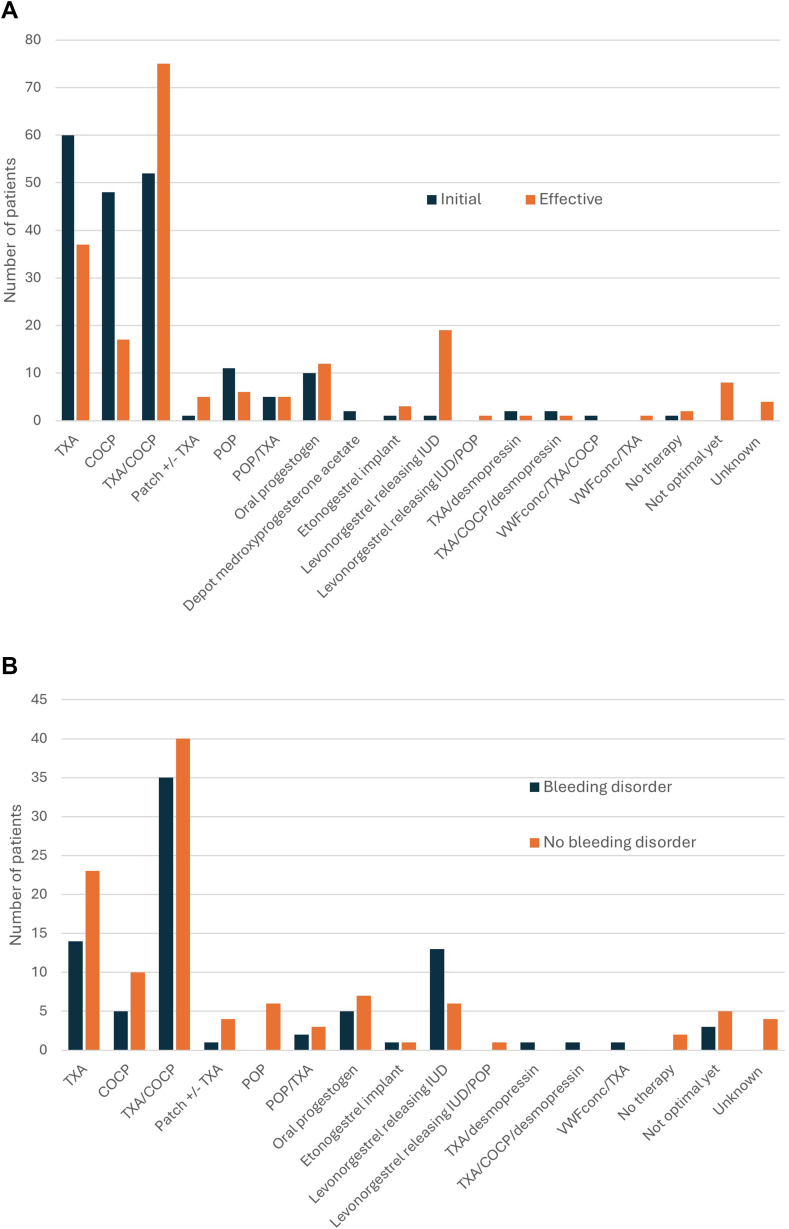
Management of heavy menstrual bleeding. (A) Initial and effective management, and (B) effective management in patients with and without bleeding disorders. COCP, combined oral contraceptive pill; IUD, intrauterine device; POP, progesterone-only pill; TXA, tranexamic acid; VWFconc, von Willebrand factor concentrate.

In most patients, the initial therapy of HMB consisted of tranexamic acid alone (*n* = 60, 30%), combined oral contraceptive pill (COCP) and tranexamic acid (*n* = 52, 26%), or COCP alone (*n* = 48, 24%; Figure 3A). In 60 patients (30%), initial therapy was successful. Change of therapy was needed once, twice, or more than twice in 28%, 19%, and 23% of the patients, respectively, to provide relief from HMB-related symptoms with minimal side effects from administered medication. Patients were followed for a median period of 14 months (IQR, 24). Effective HMB management was most frequently achieved by a combination of tranexamic acid and COCP (*n* = 75, 38%), followed by tranexamic acid alone (*n* = 37, 19%) and levonorgestrel-releasing intrauterine device (*n* = 19, 10%; Figure 3A). None of the patients with a combination of tranexamic acid and COCP developed thromboembolic disease. No large differences between optimal HMB treatment were observed in patients with or without BDs (Figure 3B).

In 8 (4%) patients, treatment for HMB was not optimal after a median follow-up of 23 months. Three of them had VWD, and 2 patients had endometriosis. Two patients did not want to start hormonal therapy.

### 3.4 Iron deficiency and anemia

Iron deficiency was present in 86 of 166 (52%) tested patients. Forty-six patients had iron-deficient anemia. Self-reported fatigue, dizziness, and headache were present in 77%, 63%, and 32% of women with iron-deficient anemia, respectively, and in 71%, 42%, and 38% of women with iron deficiency without anemia. Twelve patients (14%) with iron deficiency (including 5 patients with anemia) did not complain about fatigue, dizziness, or headache. A red blood cell transfusion was given to 9 patients with severe anemia. Iron supplementation was started in 77 patients: orally in 51 patients, intravenously in 4 patients, and a combination of oral and intravenous iron supplementation in 22 patients. An iron-rich diet was advised to only 8 patients with mild iron deficiency without symptoms.

## 4 Discussion

In a tertiary care cohort of 200 women aged ≤25 years with HMB, 24% had a known bleeding disorder at their first consultation, and in 32 of 153 (21%) remaining patients, a bleeding disorder was newly diagnosed, with VWD being the most common. HMB was the first symptom of a newly diagnosed bleeding disorder in about one-third of the patients. HMB management was successful with initial therapy in 30% of women, while effective management most often consisted of tranexamic acid in combination with COCP. Iron deficiency, with or without anemia, frequently occurred in women with HMB (52%), even in those without complaints of fatigue, dizziness, or headache.

In our study, the prevalence of newly diagnosed BDs in patients with HMB was 21%, closely aligning with the 30% prevalence recently reported in the meta-analysis by Comishen et al. [6]. A bleeding disorder should always be considered, particularly in adolescents, as young age appears to be a key factor [6]. The presence of additional bleeding symptoms, which elevate the ISTH-BAT score, further supports the need for evaluation. In our study, patients with newly diagnosed BDs were more likely to have ISTH-BAT scores of ≥5 compared with those without. However, coagulation tests should also be considered in women with HMB as their sole bleeding symptom, as HMB can be the first manifestation of a bleeding disorder. This is consistent with our findings and previous studies, where HMB was the only reported symptom in approximately 50% of patients with BDs [7,19]. Early identification of a bleeding disorder is crucial not only for managing HMB but also for ensuring appropriate care during major trauma, surgeries, and childbirth. Additionally, a recent study highlighted that while HMB can be an isolating and distressing experience for adolescents, receiving a bleeding disorder diagnosis fostered identity formation and empowerment [20].

Treatment options for HMB in young women are usually medical and depend on age, underlying (bleeding) disorder, the severity of bleeding, menstrual pattern, patients’ (and families’) acceptability of treatment options, side effects, and contraception wishes. There are several hemostatic and hormonal options available [23]. In our clinic, all options are discussed with the patient (and sometimes parent), and the final choice is based on shared decision-making. Many young women were reluctant to initiate hormonal therapy. In general, initial therapy consisted of tranexamic acid with or without hormonal therapy. The general physician usually started with COCP monotherapy before the consultation at our clinic. As a consequence, the initial therapy varied in our study. Effective HMB management was mainly achieved by a combination of tranexamic acid and COCP. This was also seen in the study of Alaqzam et al. [24]. In that study, firstline therapy consisted of hormonal therapy only, as this was directed by the gynecologist. In patients with hormonal therapy failure, addition of tranexamic acid led to 100% effectivity. Furthermore, in the study of Dowlut-McElroy et al. [25], combination therapy consisting of hormonal therapy and nonhormonal therapy (tranexamic acid with or without desmopressin) was more successful than hormonal therapy alone in young women with BDs. They recommended giving a combination of nonhormonal and hormonal modalities for treatment of HMB. However, selecting the best hormonal therapy for controlling HMB is challenging [26]. Hormonal therapy is associated with several side effects, including mood changes, spotting, headaches, growth restriction, and nausea. About 70% of our patients required multiple management strategies prior to achieving effective therapy with minimal side effects. Only a few patients were treated with the levonorgestrel-releasing intrauterine system, although its effectiveness has been shown in multiple studies [27,28]. Most young patients considered the levonorgestrel-releasing intrauterine system too invasive.

Interestingly, almost one-fifth of the patients had relief from their HMB complaints with tranexamic acid alone. Hence, tranexamic acid alone might be an initial option for younger patients who do not prefer to start with hormonal therapy and for those who are shortly after menarche and want to prevent reduction in growth. A recent study of 57 patients showed a mean absolute growth reduction of 1.8 cm at 24 months in adolescent women with HMB treated with COCP within 3 months of menarche compared with the control group [29]. Progesterone-only methods also did not affect growth and might be added to tranexamic acid if tranexamic acid alone is not effective for HMB management in young women shortly after menarche.

Adolescents are at an increased risk of iron deficiency because of menstrual blood loss, limited iron intake, and rapid growth [30]. As in other studies, iron deficiency was common in our cohort of young women with HMB [9,24,31]. More than 40% of all patients had iron deficiency, causing anemia in >50% of these patients. Almost 15% of all iron-deficient patients did not report fatigue, dizziness, or headache, the most common symptoms of iron deficiency with or without anemia. Hence, screening for iron deficiency and anemia should be done in all patients visiting the HMB clinic. Treatment of iron deficiency with oral or intravenous iron is an important element of managing women with HMB, as it contributes to improvement of quality of life [32].

This study has limitations due to its retrospective design, including missing data. Although all women were tested for VWD, some were not tested for platelet function disorders. However, additional testing might have led to an even higher frequency of BDs in this cohort. On the other hand, some patients with VWF levels between 0.30 and 0.50 IU/mL will not have true VWD, as HMB might be the result of ovulatory dysfunction alone. Furthermore, the retrospective design did not allow for optimal study of the most effective method for menstrual suppression in patients with or without BDs, but it did recognize a trend. Additionally, anemia and iron studies were not performed in all patients. As iron deficiency can be asymptomatic, the number of iron-deficient patients might, therefore, be higher. Although patients were asked to complete the PBAC score after start of treatment, only 9 post-HMB treatment PBAC scores were available in this study. Bias may have occurred, as patients with successful HMB management might be more willing to provide PBAC scores after treatment. Finally, lack of quality of life measurements is another limitation of this study, as quality of life is an important item in the broader definition of HMB.

In conclusion, HMB can be the first symptom of a bleeding disorder. Managing HMB remains complex, but combining tranexamic acid with hormonal therapy seems to be effective. All patients with HMB should be screened for iron deficiency. Given these challenges, multidisciplinary clinics bring experts together to offer personalized treatments that reduce side effects and align with patient preferences [16,26].
